# Fatal fulminant hemolysis-associated pulmonary embolism in mixed-type autoimmune hemolytic anemia: A case report

**DOI:** 10.1097/MD.0000000000018984

**Published:** 2020-02-07

**Authors:** Osamu Imataki, Kikuo Iseki, Shumpei Uchida, Makiko Uemura, Norimitsu Kadowaki

**Affiliations:** a Division of Hematology, Department of Internal Medicine, Faculty of Medicine, Kagawa University; b Division of Transfusion Medicine, Kagawa University Hospital, Kagawa, Japan.

**Keywords:** autoimmune hemolytic anemia, immune thrombocytopenic purpura, pulmonary embolism, thromboembolism, tolerance loss

## Abstract

**Rationale:**

Autoimmune hemolytic AQ5 anemia (AIHA) is an immune disorder caused by antibodies directed against unmodified autologous red blood cells. In rare cases, AIHA is comorbid with other immunological disorders; for instance, when AIHA is complicated with immunologic thrombocytopenic purpura (ITP) it is called Evans Syndrome (ES). These multiple autoimmune mechanisms are referred to as “immunological tolerance loss,” which is known as a characteristic autoimmunity specific for AIHA. And there are no estimation of the risk for thromboembolism in the “immunological tolerance loss” case.

**Patient concerns:**

A 66-year-old man was diagnosed with ES after autologous stem cell transplantation for malignant lymphoma. His background immunological status was complicated because AIHA was mixed-type (warm and cold antibody type). The direct/indirect Coombs tests were positive. The anticomplement antibody was positive and his cold hemagglutinin level had increased. Anticardiolipin antibodies were negative: anticardiolipin β2GPI antibody ≤1.2 U/mL (<3.5), anticardiolipin immunoglobulin G antibody ≤8 U/mL (<10), and anticardiolipin immunoglobulin M antibody ≤5 U/mL (<8).

**Diagnoses:**

ITP and mixed-type AIHA.

**Interventions:**

The patient achieved complete response by initial prednisolone therapy; however, he did not respond to corticosteroid therapy after AIHA recurrence. He required the red blood cell transfusion due to the progression of hemolytic anemia.

**Outcomes:**

On the fourth day of refractory treatment following AIHA recurrence, the patient had acute respiratory failure with severe hypoxia and died. The cause of death was identified as pulmonary embolism (PE) based on the laboratory data and echocardiography findings, and a literature search suggested rapidly progressive hemolysis-induced PE.

**Lessons:**

Although infrequent, comorbid thromboembolism to AIHA is well documented; however, a mixed-type AIHA case complicated with thromboembolism has not been previously reported. The combined pathophysiology of AIHA and thromboembolism should be considered in the clinical course of hemolysis. Our case suggested multiple immunological background, ITP, and mixed type AIHA, could be associated to a risk for thromboembolism (TE).

## 1 Introduction

Autoimmune hemolytic anemia (AIHA) is anemia caused by autoantibody-associated hemolysis. Sometimes, it can be complicated with immune thrombocytopenia (ITP) and is called Evans Syndrome (ES). AIHA, ITP, and ES are all autoimmune diseases based on the production of autoantibodies. In AIHA, functional abnormalities of both B and T cells have been investigated as various etiological mechanisms for this hematological disease.^[1,2]^ These abnormalities are referred to as “immunological tolerance loss,” which is known as a characteristic autoimmunity specific for AIHA.^[2]^ Considering these pathogeneses, multiple autoimmune diseases can be comorbid to AIHA.

The risk of thromboembolism (TE) (usually venous) can be estimated because the hemolytic-associated coagulopathy is attributed to common mechanisms.^[3,4]^ Regardless of the underlying disease, any hemolytic diseases can cause TE.^[3,4]^

We treated an aggressive case of acute phase mixed-type AIHA (warm-type and cold-type AIHA), which occurred on the background of multiple immunological diseases, ITP, and primary biliary cirrhosis. The patient suffered from severe pulmonary embolism (PE) during the treatment course. Here we discuss how estimating the risk of hemolysis-associated thromboembolism can avoid the fatal consequence.

## 2 Case presentation

**Figure 1:**
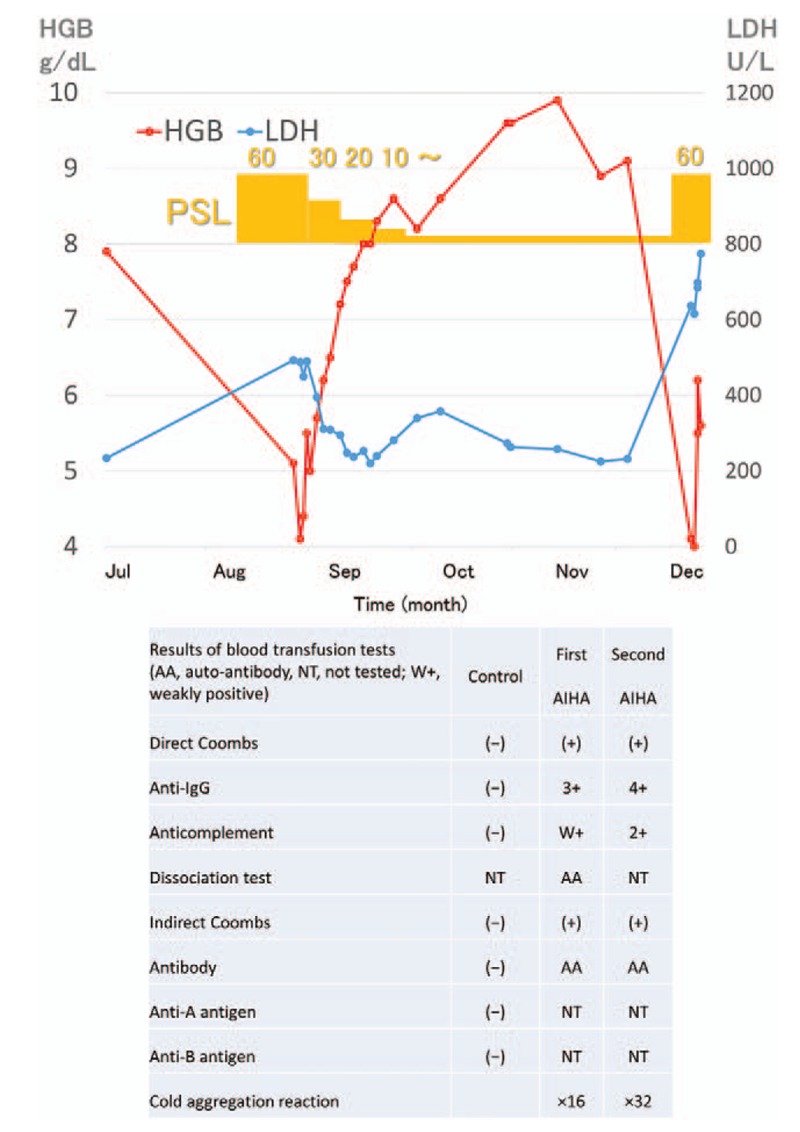
Patient's treatment course hemoglobin and lactate dehydrogenase reversely transited in the clinical course. HGB = hemoglobin, LDH = lactate dehydrogenase, PSL = prednisolone, RBC = red blood cell transfusion 2U.

A 66-year-old man was diagnosed with AIHA 1 year after undergoing autologous stem cell transplantation for relapsed malignant lymphoma, diffuse large B cell lymphoma. He was initially diagnosed with malignant lymphoma on 62-years old, then he received 8 courses of combination chemotherapy consisting of rituximab, cyclophosphamide, doxorubicin, vincristine, and prednisolone (R-CHOP). He tentatively obtained his disease remission for 4 years. After first relapse of his disease, he received salvage chemotherapy consisting of rituximab, dexamethasone, etopiside, ifosphamide, and carboplatin (R-DeVIC). Upon his second complete remission, he received autologous peripheral blood stem cell transplantation with the preparation regimen, M-BEAM.^[5,6]^ He had not been treated with immunosuppressant. His malignant lymphoma remained in remission after transplantation. He also had a 20-year history of primary biliary cirrhosis. A diagnosis of warm-antibody AIHA was made based on the following diagnostic criteria: hemolytic anemia findings based on low hemoglobin levels, elevated reticulocytes, increased indirect bilirubin levels, and decreased serum haptoglobin levels and positive direct/indirect Coombs tests (Table 1). Additionally, the patient's platelet count was below 1.0 × 10^4^/μL with the presence of antiplatelet antibodies. Bone marrow aspiration revealed an increased megakaryocyte level of 50/μL. Anti-*Helicobacter pylori* antibody was negative. We diagnosed the case as AIHA complicated with idiopathic thrombocytopenic purpura (ITP). We treated the patient for ES with 1.0 mg/kg prednisolone and maintained him on 10 mg/body prednisolone; the patient went into remission for 2 months (Fig. 1).

**Table 1:**
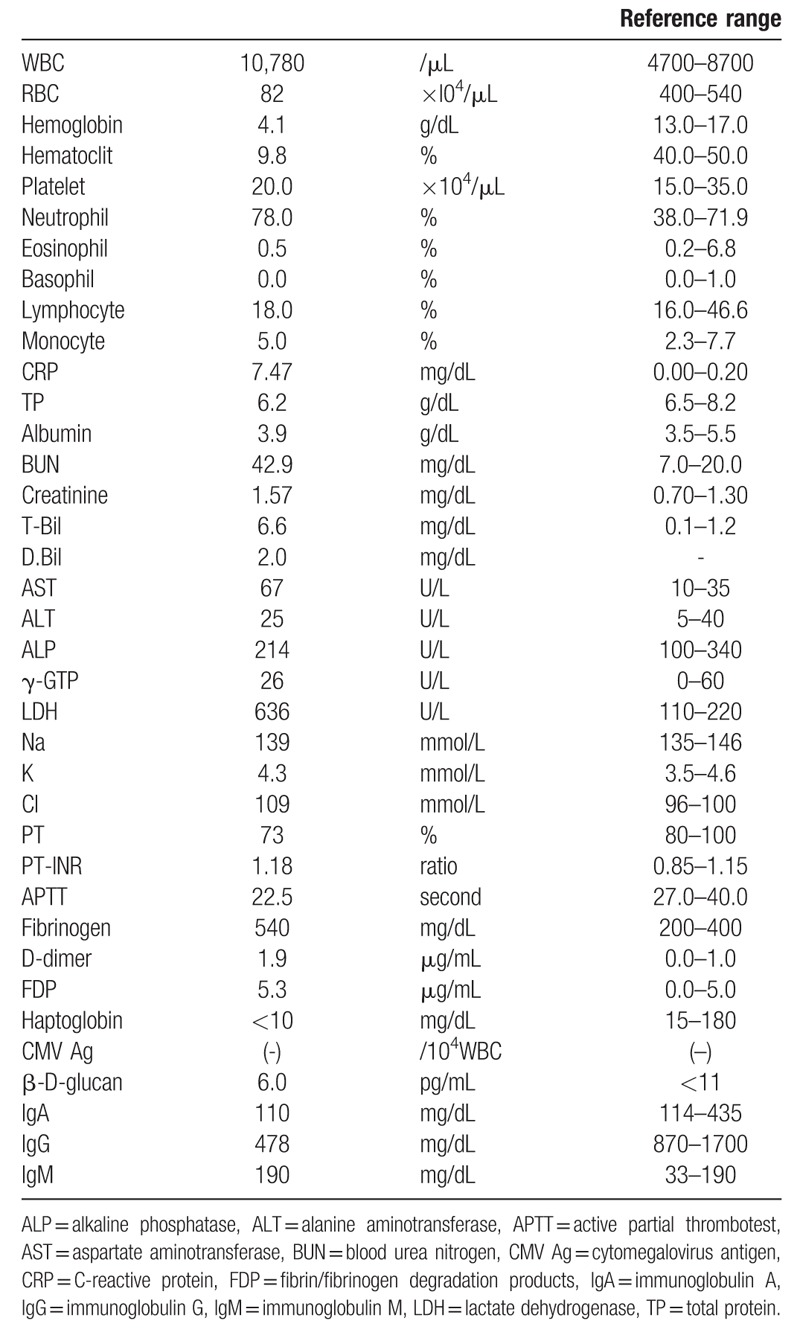
Laboratory data at the onset of initial autoimmune hemolytic anemia.

Two months after remission of AIHA, the patient's hemolysis recurred with symptoms of enterocolitis. At the second onset of AIHA, the anticomplement antibody was positive and his cold hemagglutinin level (performed at 24 °C, immunoglobulin M [IgM] type) had increased (Table 2). Donath–Landsteiner antibody was negative. Coombs tests were performed under the condition as followings; the direct antiglobulin test (DAT) and indirect antiglobulin test (IAT) were performed in ambient temperature. DAT reflected that rabbit anti-human immunoglobulin G (IgG) globulin/anti-human complement (C3b and C3d) globulin cause direct erythrocyte agglutination, IAT detect the presence of anti-erythrocyte IgG antibodies in serum. Both the direct and indirect Coombs tests were constitutively positive during his clinical course (Table 2). At the relapse of AIHA, additional antibodies were tested. However, anticardiolipin antibodies were negative: anticardiolipin β2GPI antibody ≤1.2 U/mL (<3.5), anticardiolipin IgG antibody ≤8 U/mL (<10), and anticardiolipin immunoglobulin M antibody ≤5 U/mL (<8). We diagnosed the patient with mixed-type AIHA. The second treatment with prednisolone for recurring AIHA did not decrease the hemolytic reaction. His urine and withdrawn serum were colored. He required the unwashed red blood cell transfusion due to the progression of hemolytic anemia. On the fourth day of this recurrent course of AIHA, sudden hypoxia with fulminant hemolysis resulted in respiratory distress. Echocardiography revealed an enlarged right ventricle, and the elevated tricuspid regurgitation pressure gradient was 20 mmHg. Blood tests showed coagulopathy with fibrinolysis, fibrin/fibrinogen degradation products (FDP) of 26.1 mg/mL (0–5), and a d-dimer level of 11.3 mg/mL (0–1). We diagnosed him with acute pulmonary thrombosis. The patient died of acute cardiopulmonary arrest.

**Table 2:**
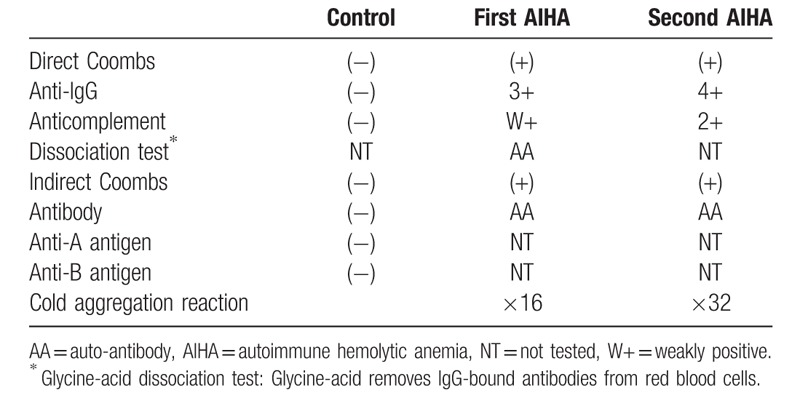
Results of blood transfusion tests during the patient's clinical course.

## 3 Discussion

This case highlights 2 essential cautions concerning AIHA. First, pulmonary thromboembolism is a common comorbid event.^[4]^ Second, there is a possibility of multiple autoimmune episodes indicated by the unique immunological etiology of AIHA.^[1,2]^ Further, these 2 conditions are mutually associated. Of note, anticardiolipin antibody is reported to be positive among a substantial portion of patients with AIHA complicated with TE.^[7]^ However, in our case, anticardiolipin antibody was negative. This suggests that multiple factors enhancing coagulopathy may commonly exist.

As shown in Table 3, a vast range of hemolytic diseases are associated with thromboembolism.^[4]^ Although the precise mechanism of thromboembolism is still under investigation, a variety of pathways may trigger hemolysis and enhance coagulopathy. The most initial step is the release of free heme from erythrocytes. Heme induces nitric oxide (NO) decrease, reactivates oxygen species, forms neutrophil extracellular traps, activates macrophages, and increases cell adhesion molecules on endothelium cells. Thus, hemolysis affects many cell types and may cause hypercoagulopathy. A review indicates that anticardiolipin antibody is enhanced in some cases of AIHA-associated PE.^[7,8]^ Based on the mechanism linking hemolysis and thromboembolism, eculizumab may be effective for prophylaxis of hemolysis-associated thromboembolism.^[9]^ A precautionary intervention could prevent the onset of thromboembolism; however, there is no standard method for this situation.^[4]^ The risk of venous thromboembolism related to warm autoimmune hemolytic anemia were investigated in a case–control study.^[10]^ According to this study, the majority of venous thromboembolism occurred during severe hemolytic flares. Other study indicated that the thromboembolism risk was associated with Hb levels ≤6 g/dL at onset, intravascular hemolysis, and previous splenectomy.^[11]^ The risk of thromboembolism increases up to 15% in the acute phase of AIHA.^[7,12]^ Thus, prophylactic anticoagulation therapy would be reasonable for patients with marked hemolysis. However, there are no standard prophylaxis regimen. The clinical benefit of heparin should be tested for preventing thromboembolism in selected cases.^[12]^

**Table 3:**
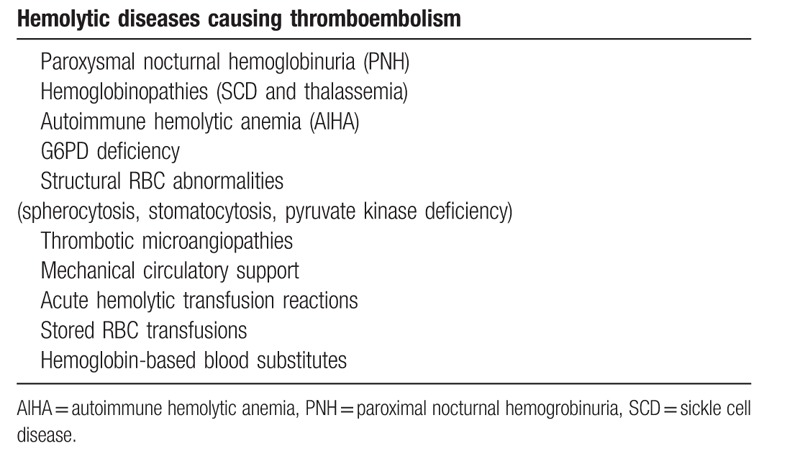
Various hemolytic diseases that may give rise to thromboembolism.

Lastly, we discuss tolerance loss in AIHA. The mechanisms of multiple immunological errors in AIHA were summarized a literature review as follows: ignored or cryptic self-antigen, polyclonal T and/or B cell activation, molecular mimicry between self- and foreign-antigens, errors in central or peripheral tolerance, and immunoregulatory disorders.^[2]^ In our patient, a stepwise exacerbation of immunological disorders was observed, including past history of primary biliary cirrhosis, warm-type AIHA, ITP, and cold-type AIHA. This might be a consequence of immunological disorders. As seen in this case, it can be difficult to treat AIHA with a background of complex immunological disorders.^[1]^ Even in simple AIHA cases, the recurrence rate is as high as 50%. Maintenance therapy with corticosteroid is thus important or remission will not be maintained.^[4]^

In conclusion, we treated a case of mixed-type AIHA and failed to treat PE during the acute hemolytic phase. A multiple immunological background could be associated to increased risk for TE.

## Author contributions

**Conceptualization:** Osamu Imataki, Makiko Uemura.

**Data curation:** Osamu Imataki, Makiko Uemura.

**Formal analysis:** Osamu Imataki, Shumpei Uchida.

**Funding acquisition:** Osamu Imataki, Makiko Uemura.

**Investigation:** Osamu Imataki, Makiko Uemura.

**Methodology:** Osamu Imataki, Kikuo Iseki, Makiko Uemura.

**Project administration:** Osamu Imataki, Makiko Uemura.

**Resources:** Osamu Imataki, Makiko Uemura.

**Supervision:** Makiko Uemura, Norimitsu Kadowaki.

**Validation:** Osamu Imataki, Makiko Uemura.

**Visualization:** Osamu Imataki, Makiko Uemura.

**Writing – original draft:** Osamu Imataki.

**Writing – review & editing:** Osamu Imataki.

Osamu Imataki orcid: 0000-0001-5332-1316.
